# The Effect of the Vesical Adaptation Response to Diuresis on Lower Urinary Tract Symptoms after Robot-Assisted Laparoscopic Radical Prostatectomy: A Pilot Proof of Concept Study

**DOI:** 10.1371/journal.pone.0159514

**Published:** 2016-07-22

**Authors:** Nobuhiro Haga, Ken Aikawa, Seiji Hoshi, Michihiro Yabe, Hidenori Akaihata, Junya Hata, Yuichi Sato, Soichiro Ogawa, Kei Ishibashi, Yoshiyuki Kojima

**Affiliations:** Departments of Urology, Fukushima Medical University School of Medicine, Fukushima, Japan; King's College London, UNITED KINGDOM

## Abstract

**Background:**

When urine output increases, voided volume at each voiding also increases in normal subjects. This is generally understood as a vesical adaptation response to diuresis (VARD). Because lower urinary tract symptoms (LUTS) are supposed to be improved by the change in bladder function after robot-assisted laparoscopic radical prostatectomy (RARP), the aim of the present study was to investigate whether VARD is involved in the improvement of LUTS after RARP.

**Methods:**

100 consecutive patients who underwent RARP and had the International Prostate Symptom Score (IPSS), quality of life (QOL) index, a frequency-volume chart (FVC), uroflowmetry, and post-voided residual urine (PVR) available were evaluated before and after RARP. This cohort was divided into patients with and without preoperative LUTS according to the preoperative IPSS total score. VARD was defined as the presence of a significant correlation between the urine output rate and voided volume at each voiding (R^2^>0.2).

**Results:**

In patients with preoperative LUTS, the IPSS total, storage, and voiding symptom scores were significantly improved after RARP (all P<0.001). The QOL index was also significantly improved after RARP (P<0.05). Although VARD was not seen before RARP (R^2^ = 0.05), it was seen after RARP (3 months R^2^ = 0.22, 12 months R^2^ = 0.23). PVR was significantly reduced after RARP (P = 0.004).

**Conclusions:**

Improvement of LUTS was seen with acquisition of VARD after RARP. As a result, urinary QOL was also improved in patients with preoperative LUTS. RARP might be an effective procedure for amelioration of LUTS by the acquisition of VARD.

## Introduction

Robot-assisted surgery performed by the Da Vinci surgical system (Intuitive Surgical, Sunnyvale, CA) provides both clear 3-dimensional vision of the surgical field and meticulous maneuvering using robot arms, leading to high-precision surgery. Thus, much excellent data regarding oncological and functional outcomes of robot-assisted radical prostatectomy (RARP) have been gathered.[1–4] While RARP provides earlier acquisition of urinary continence than other surgical approaches for radical prostatectomy,[1–4] recent attention has shifted the focus to the effect of RARP on lower urinary tract function and lower urinary tract symptoms (LUTS) excluding urinary incontinence.[5–8]

Our institution has shown, from frequency-volume chart (FVC) analyses, the interesting phenomenon of detrusor activity changes in response to diuresis in experimental and clinical studies.[9–12] The phenomenon is that high diuresis increases the voided volume at each voiding, and low diuresis decreases the voided volume at each voiding. Thus, voided volume at each void is constantly changing according to diuresis. We called this phenomenon the “vesical adaptation response to diuresis (VARD)”. On the other hand, this response is lacking in pathological conditions, such as overactive bladder.[10]

Although patients who undergo radical prostatectomy have preoperative LUTS at a relatively constant rate, LUTS gradually improves with time after radical prostatectomy.[13–15] However, the mechanism of improvement of LUTS after radical prostatectomy has not been fully clarified to date. Because preoperative LUTS was supposed to improve in association with the amelioration of bladder function after radical prostatectomy, we hypothesized that VARD is involved in the improvement of LUTS after radical prostatectomy.

Because the association between VARD and the amelioration of LUTS after RARP has not yet been investigated, this study was conducted as a pilot proof of principle study. The aim of this pilot study was to determine whether VARD was present before and after RARP. If VARD occurred before and/or after RARP, the involvement of VARD in LUTS was evaluated.

## Materials and Methods

The participants in this prospective clinical cohort observational study were 100 consecutive patients who underwent RARP at our institution between February 2013 and November 2013. To investigate the effect of VARD on LUTS, these patients were divided into the two groups, with and without preoperative LUTS according to the International Prostate Symptom Score (IPSS) before RARP. Preoperative LUTS was defined as an IPSS total score greater than 8. In the two groups, no patients had baseline lower urinary tract abnormalities, such as neurogenic bladder. No patients underwent salvage radiotherapy, took medications that affected lower urinary tract function, such as alpha blockers or antimuscarinics, or underwent bladder neck contracture or urethral stricture surgeries or injectable treatments, such as collagen into the membranous urethra, for the duration of postoperative evaluation of urinary function. Written, informed consent was obtained from all patients before the study after explaining its purpose and methods. The study protocols were approved by the ethics committee of Fukushima Medical University (clinical trial registration number 2334).

### Operative technique

All cases underwent surgery using the 3-arm Da Vinci Si surgical system (Intuitive Surgical, Sunnyvale, CA) with combined posterior and anterior intraperitoneal approaches and early exposure of the seminal vesicles and vasa deferentia. In all cases, the anastomosis between the urethra and bladder started with the Rocco technique for posterior reconstruction of Denonvilliers’ fascia,[16] followed by Van Velthoven’s stitch[17] using a running, double-armed, barbed 3–0 polyglyconate suture (V-LOC^®^; Covidien, Mansfield, MA). However, anterior reconstruction was not performed in this cohort. Integrity of the urethrovesical anastomosis was confirmed intraoperatively with intravesical instillation of 150 mL of sterile saline. RARP was performed or supervised by a single surgeon (Y.K.).

### Evaluation of LUTS and urinary function before and after RARP

LUTS was evaluated using the IPSS and a quality of life (QOL) index. Moreover, the IPSS subscores, such as the voiding symptom score (the sum of the intermittency score, weak stream score, and straining score) and the storage symptom score (the sum of the frequency score, urgency score, and nocturia score), were also assessed as individual scores.[18]

To assess lower urinary tract function, patients were asked to write down the 24-h FVC, which recorded the volumes voided, as well as the time of each micturition.[19] In the present study, in order to focus on the voiding behavior during the awake period, data while patients were asleep were not evaluated, since maximum bladder capacity while in bed was increased in normal subjects.[20] On FVCs, the following were evaluated: 1) voided volume at each voiding (mL); 2) maximum voided volume during daytime (mL); 3) micturition frequency (/wake time); 4) urine volume during the awake period (mL); and 5) urine output rate (mL/min). Urine volume during the awake period included the last void before going to bed but excluded the first void after rising.[19] The urine output rate was calculated by dividing the volume voided by the interval between 2 successive micturitions. VARD was defined as the presence of a significant correlation between the urine output rate and voided volume at each voiding (R^2^>0.2). Lower urinary tract function was also evaluated using uroflowmetry (UFM) and post-void residual urine volume (PVR) as determined by ultrasonography.

Urinary incontinence was assessed by two methods. First, subjective evaluation of urinary continence was based on the patient using either no pads or only one security liner during stressful activities.[21] The second item was a 1-h pad test.[22] This 1-h pad test was performed according to International Continence Society recommendations, with a bladder volume >200 ml as evaluated by ultrasound. This bladder volume was achieved by natural diuresis following ingestion of 500 ml of water. The patient performed manoeuvers and exercises during the 1-h period while wearing a pre-weighed absorbent pad, which was again weighed at the completion of provocation exercises to calculate urine loss. Urinary continence on the 1-h pad test was defined as <2 ml.[22] Thus, urinary continence was judged as present if both requirements were met.

LUTS and lower urinary tract function were assessed just before RARP and then at 3 and 12 months after RARP. The goal of this prospective study was to investigate whether VARD had occurred before and/or after RARP, and then, to investigate the effect of VARD on LUTS.

### Statistical analysis

All values are presented as means ± standard deviation. The correlations between continuous variables were investigated by simple regression analysis using the square of Pearson’s correlation coefficient. To evaluate the differences in the coefficient of correlation among before RARP, 3 months, and 12 months after RARP, the equivalence test of the coefficient of correlation was performed using the following formula.

$$z=\frac{z^{\prime }{}_{1}-z^{\prime }{}_{2}}{\sqrt{\frac{1}{n_{1}-3}+\frac{1}{n_{2}-3}}}$$

Z'_1_ and z'_2_ are *r* values after *z* transformation.

However, because this formula is a comparison between two groups, Bonferroni adjustment was needed for multiple comparisons after the equivalence test of the coefficient of correlation among the three time points. *P*-values < 0.05/3 were considered significant in this equivalence test after Bonferroni adjustment.

To examine how the outcome data changed over time, linear mixed effect models were fitted. Analysis of linear mixed effect models was undertaken using SPSS version 23 software (Statistical Package for Social Sciences, Chicago, IL). All data analyses other than analyses of linear mixed effect models were performed using Ekuseru-Toukei 2012 software (Social Survey Research Information Co., Ltd., Tokyo, Japan). *P*-values < 0.05 were considered significant except for the data after Bonferroni adjustment for multiple comparisons.

## Results

A total of 100 men entered the study; their baseline characteristics and operative outcomes are shown in Table 1. There were 57 patients without preoperative LUTS and 43 patients with preoperative LUTS. In the present study, there were no significant differences in the comorbidities, i.e. hypertension, diabetes mellitus, and hyperlipidemia, between the patients with and without preoperative LUTS. Excluding the IPSS scores, there were no significant differences in patients’ characteristics or operative outcomes between the two groups.

**Table 1:** Preoperative patients’ characteristics, operative procedures, and IPSS scores.

	Median (range), Mean (±SD), and number of patients		
Variable	Patients without LUTS	Patients with LUTS	P-value
N	57	43	
Age (years)	65 (52–78)	67 (57–76)	0.13
BMI (kg/m^2^)	24 ±2	24 ±2	0.25
Comorbidity (hypertension)	23	25	0.18
Comorbidity (diabetes mellitus)	15	9	0.66
Comorbidity (hyperlipidemia)	7	6	0.69
D‘Amico risk classification (low: intermediate: high)	27: 20: 10	19: 18: 6	0.82
Operative (console) duration (min)	147 ± 38	146 ±29	0.76
EBL (mL)	345± 271	330±240	0.84
Bilateral NS: Unilateral NS: non-NS	6: 17: 34	3: 11: 29	0.52
Prostate weight (g)	44 ±15	45 ±19	0.94
Duration of catheter insertion (days)	6 ± 1	7 ± 4	0.14
Pathological stage (T2: ≥ T3)	45: 12	35: 8	0.41
Total IPSS score (preoperative)	3.4 ± 2.0	15.1± 6.3	<0.0001
Voiding symptom score (preoperative)	1.0 ±1.1	7.2 ± 4.2	<0.0001
Storage symptom score (preoperative)	2.1 ±1.4	6.2 ±3.5	<0.0001
QOL index (preoperative)	2.3 ± 1.4	3.9 ±1.9	<0.0001

LUTS, lower urinary tract symptoms; IPSS, International Prostate Symptom Score; SD, standard deviation; BMI, body mass index; EBL, estimated blood loss; NS, nerve-sparing procedure; Operative duration, duration of operation without docking/undocking the robot. Voiding symptom score is the sum of the intermittency score, weak stream score, and straining score of the IPSS; Storage symptom score is the sum of the frequency score, urgency score, and nocturia score of the IPSS; QOL, quality of life; LUTS was defined as present when the IPSS total score was greater than 8.

In the present study, 28 patients underwent a unilateral nerve-sparing (NS) procedure, and nine patients underwent a bilateral NS procedure. There were no significant differences between the unilateral and bilateral NS groups in the postoperative IPSS, QOL index, 1-h pad test, maximum flow rate on uroflowmetry, and PVR. (S1 Table) In addition, there were no significant correlations between the QOL index score and prostate size before and after RARP (before RARP R^2^ = 0.003, 3 months after RARP R^2^ = 0.004, 12 months after RARP R^2^ = 0.014) in the present study.

Longitudinal changes of the number of incontinent patients and several parameters of IPSS, uroflowmetry, and FVCs are shown in both Table 2 (patients without preoperative LUTS) and Table 3 (patients with preoperative LUTS).

**Table 2:** Longitudinal changes of several parameters in patients without preoperative LUTS.

	Mean (±SD) or number of patients				
Variable	Before RARP	3 mo. after RARP	P	12 mo. after RARP	P
Incontinent patients (%)	-	37		10	
Pad counts in incontinent patients	-	1.1 ± 1		0.5 ± 0.7	
1-h pad test in incontinent patients	-	12 ±26		6 ± 26	
Total IPSS score	3.4 ± 2.0	7.1 ±5.2*	p<0.01	3.7 ±3.6*	p<0.01
Voiding symptom score	1.0 ± 1.1	2.2±3.1*	p<0.01	0.9 ± 1.9	p = 0.84
Storage symptom score	2.1 ± 1.4	4.1 ± 2.3*	P<0.01	2.2 ± 1.5	p = 0.81
QOL index	2.3 ± 1.4	2.8 ± 1.9	p = 0.51	2.2± 1.4	p = 0.08
Maximum flow rate in UFM (mL/sec)	18 ±6	17 ±11	p = 0.87	18± 10	p = 0.91
PVR (mL)	37 ± 38	23 ±18*	P<0.01	23 ± 18*	P<0.01
MVV for the awake period (mL)	351± 88	249 ± 109*	P<0.01	284 ± 119*	P<0.01
Micturition frequency (/wake time)	6 ± 1	7 ± 2*	P<0.01	6 ± 2	p = 0.24
Urine volume during the awake period (mL)	1530 ± 457	1209 ± 557*	p = 0.01	1275 ± 505*	p<0.01

LUTS, lower urinary tract symptom; IPSS, International Prostate Symptom Score; RARP, robot-assisted laparoscopic radical prostatectomy; mo., months; SD, standard deviation; Voiding symptom score is the sum of the intermittency score, weak stream score, and straining score of the IPSS; Storage symptom score is the sum of the frequency score, urgency score, and nocturia score of the IPSS; QOL, quality of life; UFM, uroflowmetry; PVR, post-voided residual urine volume MVV, Maximum voided volume; P values indicate the comparison before RARP.

* indicates P<0.05 compared with before RARP

**Table 3:** Longitudinal changes of several parameters in patients with preoperative LUTS.

	Mean (±SD) or number of patients				
Variable	Before RARP	3 mo. after RARP		12 mo. after RARP	
Incontinent patients (%)	-	31		7	
Pad counts in incontinent patients	-	1.3 ± 0.8		0.6 ± 0.6	
1-h pad test in incontinent patients	-	16 ± 37		2 ± 2	
Total IPSS score	15.1 ±6.3	11.7 ± 8.1*	p<0.01	6.7± 5.9*	p<0.01
Voiding symptom score	7.2 ± 4.2	3.9 ± 4.0*	p<0.01	1.9 ± 3.1*	p<0.01
Storage symptom score	6.2 ±3.5	6.5 ± 3.4	p = 0.63	4.1 ±2.6*	p<0.01
QOL index	3.8 ± 1.3	3.9 ± 1.9	p = 0.80	2.8 ±1.7*	p<0.01
Maximum flow rate in UFM (mL/sec)	14 ± 7	16 ± 8	p = 0.16	17 ± 8	p = 0.07
PVR (mL)	36 ± 37	24 ± 17	p = 0.06	18± 13*	p<0.01
MVV for the awake period (mL)	295± 99	212 ± 102*	p<0.01	254 ±84*	p<0.01
Micturition frequency (/wake time)	8 ± 3	8 ± 2	p = 0.80	7 ± 2	p = 0.15
Urine volume during the awake period (mL)	1413 ± 556	1346 ± 692	p = 0.39	1389 ± 551	p = 0.69

LUTS, lower urinary tract symptom; IPSS, International Prostate Symptom Score; RARP, robot-assisted laparoscopic radical prostatectomy; mo., months; SD, standard deviation; Voiding symptom score is the sum of the intermittency score, weak stream score, and straining score of the IPSS; Storage symptom score is the sum of the frequency score, urgency score, and nocturia score of the IPSS; QOL, quality of life; UFM, uroflowmetry; PVR, post-voided residua urine volume; MVV, Maximum voided volume; P values indicate the comparison before RARP.

* indicates P<0.05 compared with before RARP.

In the present study, there were no significant differences in the ratio of incontinent patients to continent patients or the volume of urinary leakage in the incontinent patients between the groups without and with preoperative LUTS, both at 3 months and at 12 months after RARP (Tables 2 and 3) (data not shown).

**Fig 1:**
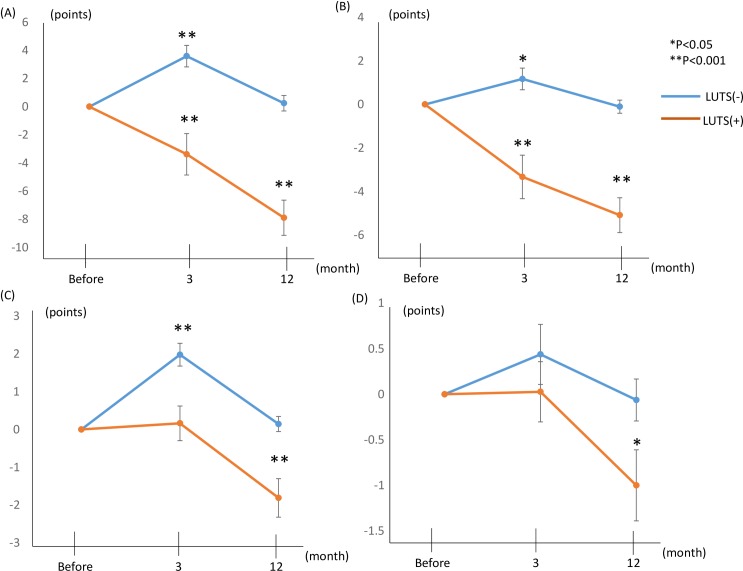
Changes from the baseline values in the International Prostate Symptom Score (IPSS) and quality of life (QOL) index in patients with and without lower urinary tract symptoms (LUTS) evaluated before robot-assisted laparoscopic radical prostatectomy. **A)** Total IPSS score. **B)** Voiding symptom score. **C)** Storage symptom score. **D)** QOL index. The voiding symptom score is the sum of the intermittency score, weak stream score, and straining score of the IPSS; the storage symptom score is the sum of the frequency score, urgency score, and nocturia score of the IPSS.

On the IPSS, the changes of the IPSS total score and subscores from the baseline values before RARP were investigated in the groups without and with preoperative LUTS (Fig 1, Tables 2 and 3). In the group without preoperative LUTS, the IPSS total score, voiding symptom score, and storage symptom score were significantly aggravated at 3 months after RARP, and these eventually returned to baseline values at 12 months after RARP (Fig 1). The QOL index was not changed between before and after RARP (Fig 1).

In the group with preoperative LUTS, the IPSS total score, voiding symptom score, and storage symptom score were not significantly aggravated at 3 months after RARP; instead, the IPSS total score and voiding symptom score were significantly improved at 3 months after RARP (Fig 1). Lastly, all scores were significantly improved at 12 months after RARP (Fig 1), as was the QOL index (Fig 1).

In addition to the IPSS scores, the changes from the baseline values before RARP of each group in several parameters of UFM and FVC were investigated (Tables 2 and 3). On UFM, the maximum flow rate was improved after RARP only in the group with preoperative LUTS, but it was not significant. PVR was significantly reduced 12 months after RARP in both groups (Tables 2 and 3). On FVC analyses, maximum voided volume was significantly reduced 3 months after RARP in both groups, but it did not return to baseline values in both groups 12 months after RARP (Tables 2 and 3). Voiding frequency was significantly increased in the group without preoperative LUTS 3 months after RARP, but it was not increased in the group with preoperative LUTS (Tables 2 and 3).

**Fig 2:**
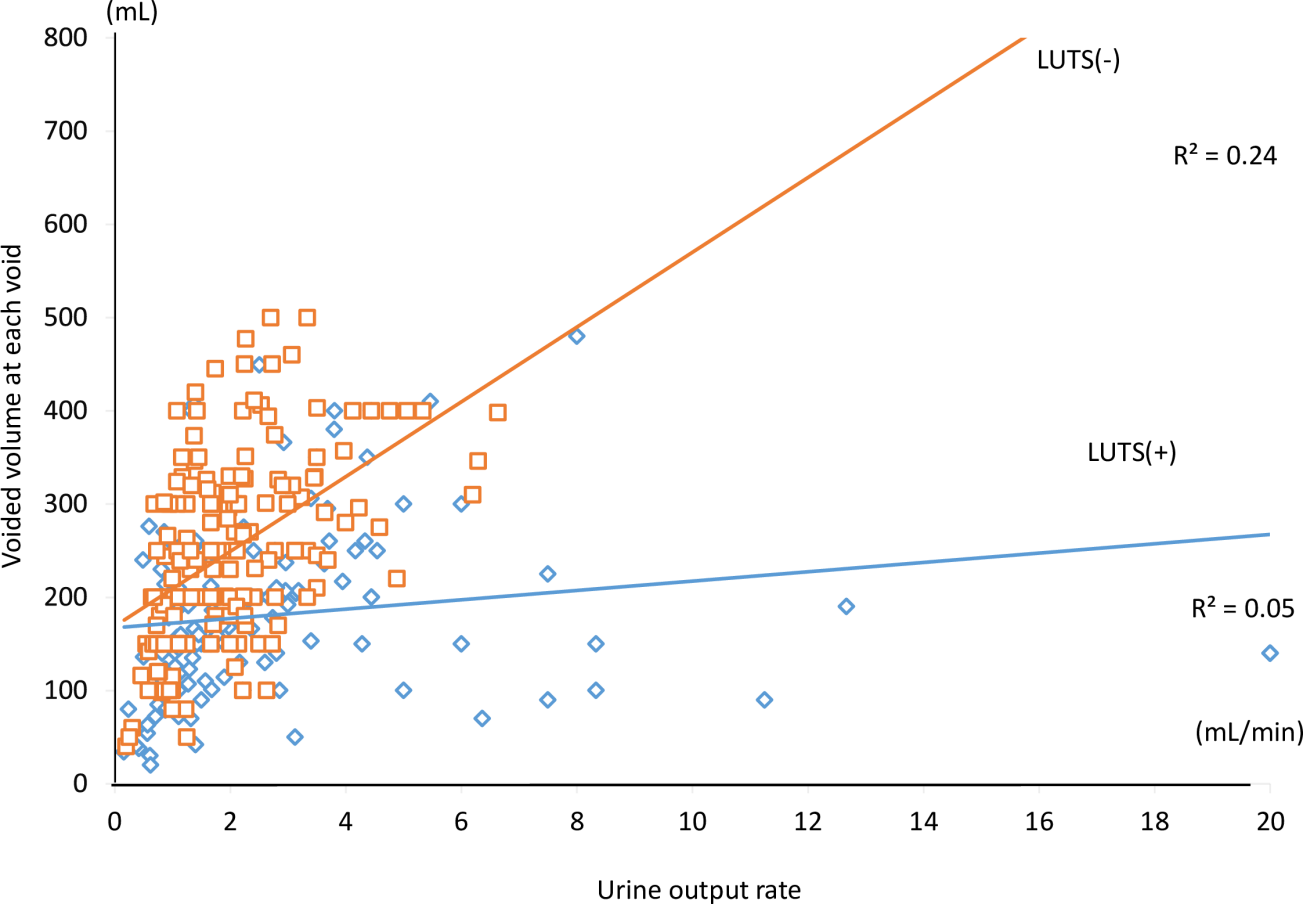
The scatter plots of the urine output rate versus the voided volume at each voiding in the patients with and without lower urinary tract symptoms (LUTS) before robot-assisted laparoscopic radical prostatectomy (RARP). Blue diamond plots and the blue regression line indicate the micturitions of the patients with preoperative LUTS. The coefficient of determination is 0.05. Red square plots and the red regression line indicate the micturitions of the patients without preoperative LUTS. The coefficient of determination is 0.24. The vesical adaptation response to diuresis is seen in patients without preoperative LUTS.

**Fig 3:**
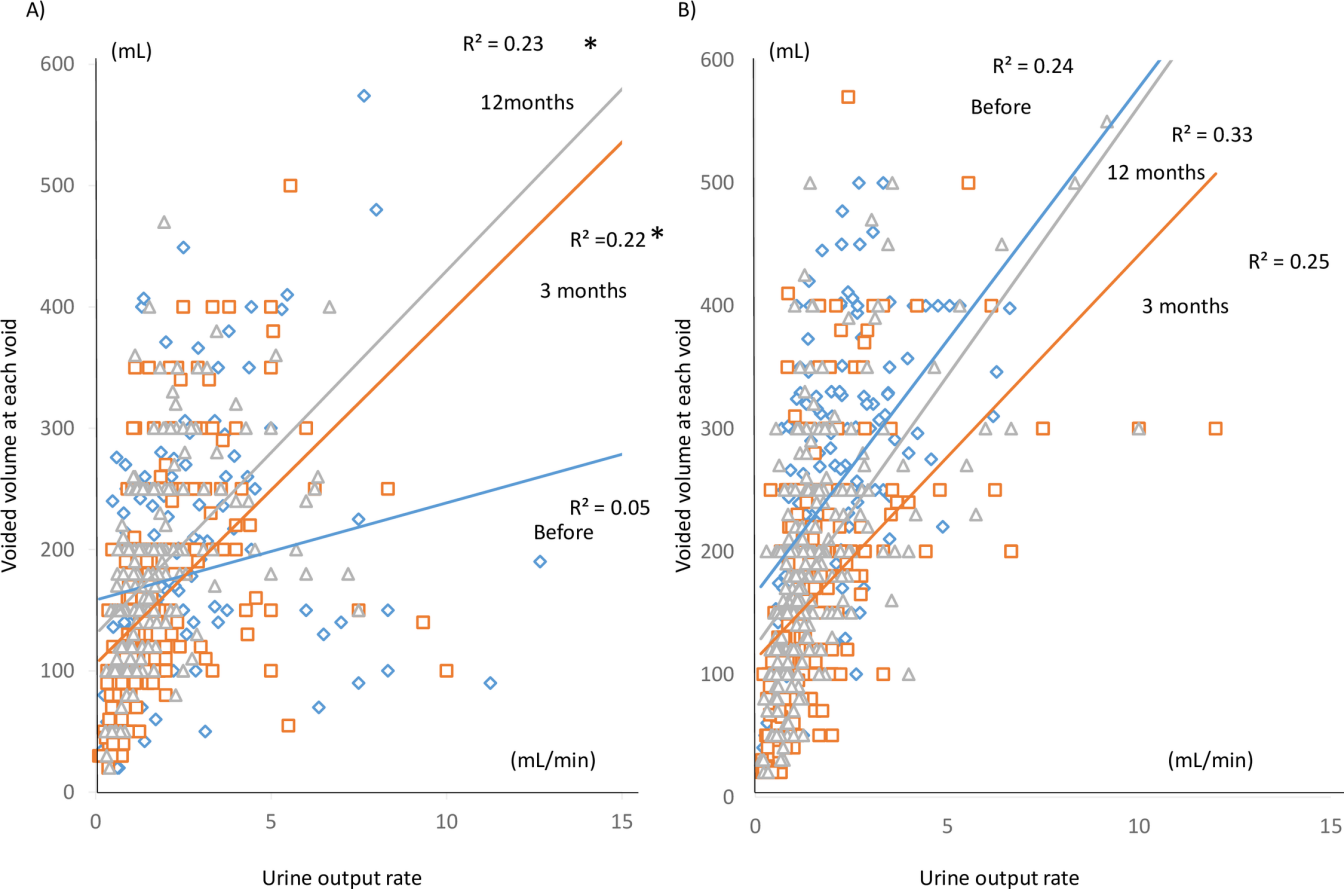
The scatter plots of the urine output rate versus the voided volume at each voiding. **A) Micturition plots of the patients with preoperative LUTS stratified by evaluation time period.** Blue diamond plots and the blue regression line indicate the micturitions before RARP. The coefficient of determination is 0.05. Orange square plots and the orange regression line indicate the micturitions 3 months after RARP. The coefficient of determination is 0.22. Gray triangle plots and the gray regression line indicate the micturitions 12 months after RARP. The coefficient of determination is 0.23. * indicates significant differences of the coefficient of correlation before RARP after Bonferroni adjustment. **B) Micturition plots of the patients without preoperative LUTS stratified by evaluation time period**. Blue diamond plots and the blue regression line indicate the micturitions before RARP. The coefficient of determination is 0.24. Orange square plots and the orange regression line indicate the micturitions 3 months after RARP. The coefficient of determination is 0.25. Gray triangle plots and the gray regression line indicate the micturitions 12 months after RARP. The coefficient of determination is 0.33.

On FVC analyses, a preliminary analysis of VARD before RARP was conducted. VARD was present in the group without preoperative LUTS, but it was not present in the group with preoperative LUTS (Fig 2). Therefore, the results of the patients not having LUTS in the present study were consistent with our previous studies.[10–12] In the group with preoperative LUTS, although VARD was not seen before RARP (R^2^ = 0.05), it was seen after RARP (3 months R^2^ = 0.22, 12 months R^2^ = 0.23; Fig 3A). In the group without preoperative LUTS, VARD was seen from the preoperative period (before RARP R^2^ = 0.24, 3 months after RARP R^2^ = 0.25, 12 months after RARP R^2^ = 0.33; Fig 3B). All data could be cited in S1 File and S2 File. Although significant differences in correlations between the urine output rate and voided volume at each voiding were observed between before and after RARP in the group with preoperative LUTS (before RARP and 3 months after RARP: z = -3.06, P = 0.002 < 0.05/3; before RARP and 12 months after RARP: z = - 2.41, P = 0.016 < 0.05/3), there were no significant differences before and after RARP in the group without preoperative LUTS (before RARP and 3 months after RARP: z = -1.99 P = 0.046 > 0.05/3; before RARP and 12 months after RARP: z = 0.35, P = 0.726 > 0.05/3). Therefore, the correlations between the urine output rate and voided volume at each voiding were not significantly changed in the group without preoperative LUTS before and after RARP.

## Discussion

The present study investigated the presence or absence of VARD after RARP and the association between VARD and LUTS/lower urinary tract function. In the group with preoperative LUTS, although VARD was not seen before RARP, it was seen after RARP. With the acquisition of VARD, PVR was significantly reduced after RARP. The IPSS total, storage, and voiding symptom scores were significantly improved after RARP. The QOL index was also significantly improved after RARP. On the other hand, in the group without preoperative LUTS, VARD was seen before RARP and was maintained during the observation period. The IPSS total score, voiding symptom score, and storage symptom score were significantly aggravated at 3 months after RARP, and these eventually returned to baseline values at 12 months after RARP. The QOL index was not changed between before and after RARP.

Several methodological limitations must be considered in the present study. First, a simple 1-day FVC rather than a longer record of voiding behavior was used. However, Fitzgerald et al. reported that a 24-h FVC was clinically useful and sufficient for the analysis of voiding behavior in women with overactive bladder,[23] and little difference was observed in the value of voiding diary variables when a 24-h diary was compared to diaries of 2 or 3 days’ duration.[24] Second, the maximum flow rate was not significantly improved after RARP in both the patients with and without preoperative LUTS in the present study. Skarecky et al. reported that the maximum flow rate on uroflowmetry improved significantly after RARP.[25] As just described, it is generally considered that the maximum flow rate after radical prostatectomy would be improved by removing the bladder outlet obstruction. The reason why the maximum flow rate was not significantly improved in the present study is that the true result might have been masked by the small sample size. Third, the appropriate NS technique could reduce postoperative urinary incontinence and detrusor overactivity. Hence, it is important to evaluate the association between VARD and the NS procedure. However, because the sample size was small in the present study, this association could not be evaluated. Further studies would be needed to elucidate this association. Last, a single surgeon or cases supervised by a single surgeon would add a further bias. There might be differences amongst surgeons in the tension of sutures for the posterior and/or anterior reconstructions despite using a V-LOC^®^ to prevent slippage of the suture, and bites and/or pinches during vesicourethral anastomosis. As a result, differences in postoperative outcomes regarding LUTS and lower urinary tract function would occur among surgeons. However, in the present study, the Da Vinci Si surgical system (Intuitive Surgical, Sunnyvale, CA), which has a dual console system, was used. Meticulous maneuvers could be accomplished with the Da Vinci Si during the operation, and fine alignment could be implemented by the experienced surgeon positioned at another console in the dual console system. Therefore, differences among surgeons in the vesicourethral anastomosis and reconstruction suture would be minimal in the present study.

To date, most studies of lower urinary tract function and LUTS before and after radical prostatectomy have used not only several questionnaires[5, 14, 15], but also urodynamic studies.[7, 8] However, evaluation of lower urinary tract function by questionnaires has been found to have discrepancies between the answers of the patients and actual voiding behavior.[26] Although urodynamic studies, such as pressure-flow studies, cystometry, abdominal leak point pressure, and so on, evaluate lower urinary tract function precisely, they have the drawbacks of both invasiveness to the patients due to catheterization and the examination performed in a non-physiological situation at the clinical site. In contrast, an FVC is considered to less-invasively provide precise information about actual voiding behavior in daily life.[20, 26] In the present study, an FVC was used to accurately assess the voiding behavior and investigate the effect of VARD on LUTS after RARP.

In the present study, the preoperative preliminary analysis demonstrated that the patients without preoperative LUTS had VARD, but the patients with preoperative LUTS did not. In our previous study, we demonstrated that OAB patients lack VARD.[10] These data suggest that the pathogenesis of LUTS is associated with lack of VARD. In addition, although VARD was not observed in patients with preoperative LUTS, VARD was seen after RARP, following the improvement of LUTS. Therefore, this phenomenon might support the association between VARD and LUTS. In other words, acquisition of VARD may improve LUTS. However, the main cause of the amelioration of voiding symptoms after RARP might be the reduction of bladder outlet obstruction caused by the removal of the prostate because of a significant decrease of PVR and improvement in the maximum flow rate. In the present study, the contributions of the acquisition of VARD and the reduction of bladder outlet obstruction in the improvement of voiding symptoms could not be evaluated. With regard to the storage symptoms and storage function, after acquisition of VARD, micturition frequency during the daytime was maintained despite the decrease in the maximum voided volume after RARP, and storage symptoms improved 12 months after RARP in the preoperative LUTS group. These findings might suggest that both storage symptoms and storage function were improved, at least in part, by acquisition of VARD, especially in the preoperative LUTS group.

In patients without preoperative LUTS, VARD was present before RARP. Although significant differences in the correlations between the urine output rate and voided volume at each voiding were not observed before and after RARP in patients without preoperative LUTS, VARD was maintained during the observational period. Although LUTS was temporarily aggravated in patients without LUTS at 3 months after RARP, LUTS eventually returned to baseline values at 12 months after RARP. Namely, maintenance of VARD may prevent the permanent aggravation of LUTS. Thus, this phenomenon also suggests the association between VARD and LUTS. The reason why LUTS was not improved after RARP might be because IPSS scores at each evaluation time point were low in patients without LUTS compared to those with LUTS.

In our previous studies, VARD was seen in normal subjects.[9–12] In addition, VARD was acquired after RARP in patients with LUTS, following the improvement of LUTS and lower urinary tract dysfunction in the present study. Therefore, VARD could be considered a physiological reaction of a living organism with normalization of lower urinary tract function. However, the acquisition mechanism of VARD has yet to be clarified.[9–12] Several reports demonstrated that prostatectomy improved bladder blood flow[27] and re-innervation of the dominant nerve of the bladder,[28] leading to improved lower urinary tract function of the bladder. Another study demonstrated that detrusor overactivity in patients with prostatic enlargement was improved by pharmacologic ablation by application of lidocaine to the prostatic urethra.[29] This experimental study suggests that abnormal sensory afferent inputs from the prostate aggravated the storage function of the bladder, and it was remedied by the pharmacological ablation of the sensory afferent neurons from the prostate. The findings both from the reported studies mentioned above and the present study would suggest that acquisition of VARD after RARP might be involved in the improvement of bladder blood flow, re-innervation of branches to the bladder, and neuromodulation of afferent nerves from the prostate. However, further clinical and experimental studies are needed to elucidate the acquisition mechanism by combining the measurement of bladder blood flow and pathological studies of the bladder and neurons from the lower urinary tract.

The present study was a proof of concept study. Therefore, a sample size calculation was not considered necessary, because the main purpose of a proof of concept study is to collect information for future confirmatory studies.

## Conclusions

Improvements of LUTS and lower urinary tract dysfunction were seen with acquisition of VARD after RARP in patients with preoperative LUTS. As a result, urinary QOL was significantly improved in patients with preoperative LUTS. VARD could be considered a physiological reaction of a living organism that occurs with normalization of lower urinary tract function. RARP might be an effective procedure for amelioration of LUTS through acquisition of VARD.

## Supporting Information

S1 File VARD IPSS more than 8. The patients’ clinical parameters, which was recorded in the patients with preoperative LUTS, were described.
(PDF)

S2 File VARD IPSS less than 8. The patients’ clinical parameters, which was recorded in the patients without preoperative LUTS, were described.
(PDF)

S1 Table Comparison of postoperative outcomes between the unilateral and billateral nerve-sparing groups. (XLSX)
