# Supplementary material for: The Effect of the Vesical Adaptation Response to Diuresis on Lower Urinary Tract Symptoms after Robot-Assisted Laparoscopic Radical Prostatectomy: A Pilot Proof of Concept Study
Source: PLoS One. 2016 Jul 22;11(7):e0159514. doi: 10.1371/journal.pone.0159514 (PMC4957788; doi:10.1371/journal.pone.0159514)
Supplement: S1 File — The patients’ clinical parameters, which was recorded in the patients with preoperative LUTS, were described. (PDF) [file pone.0159514.s001.pdf]

調査時期	利尿率	一回排尿量(術前)	一回排尿量(3m)	一回排尿量(12m)
術前	0.4	50		
術前	0.758621	110		
術前	0.666667	20		
術前	0.5	50		
術前	0.666667	50		
術前	0.842105	80		
術前	1.538462	100		
術前	0.909091	100		
術前	1.714286	60		
術前	1.538462	100		
術前	3.076923	200		
術前	1.111111	200		
術前	0.952381	200		
術前	1.111111	100		
術前	1.666667	200		
術前	0.666667	100		
術前	0.6875	110		
術前	1.105263	210		
術前	1.105882	188		
術前	1.273684	242		
術前	0.857143	120		
術前	0.846154	110		
術前	0.878571	123		
術前	1.383333	166		
術前	1.082759	157		
術前	3.4	306		
術前	3.945455	217		
術前	4.333333	260		
術前	2.317647	197		
術前	1.133333	119		
術前	2.738462	178		
術前	2.235772	275		
術前	3	192		
術前	1.618557	157		
術前	1.27381	107		
術前	1.976471	168		
術前	1.669291	212		
術前	1.1	253		
術前	1.868132	170		
術前	2.166667	130		
術前	1.766667	159		
術前	2.5	150		
術前	1.111111	200		
術前	1.5	150		
術前	4.444444	200		
術前	0.877193	250		
術前	1.666667	150		
術前	0.666667	100		
術前	1.008696	116		
術前	1.05	126		
術前	0.422222	38		
術前	1.055172	153		
術前	1.627586	236		
術前	2.364706	201		
術前	3.138462	204		
術前	1.273333	191		
術前	1.25	150		
術前	2.5	150		

術前	2.5	150
術前	0.909091	100
術前	1.176471	200
術前	1.05	126
術前	1.9	114
術前	0.503704	136
術前	1.683333	101
術前	2.371429	166
術前	3.4	153
術前	3.630769	236
術前	6	150
術前	2.6	130
術前	1.294737	123
術前	0.816327	200
術前	4.545455	250
術前	2.5	200
術前	0.833333	150
術前	2.5	150
術前	1.666667	150
術前	2.222222	200
術前	2.222222	200
術前	0.873469	214
術前	4.375	350
術前	1.315789	250
術前	4.166667	250
術前	2	300
術前	1.5625	250
術前	5	300
術前	0.75	150
術前	2.222222	200
術前	5	100
術前	0.789474	150
術前	0.833333	200
術前	0.705882	72
術前	0.568421	54
術前	0.752212	85
術前	1.107692	72
術前	1.4	42
術前	0.372727	41
術前	1.044444	141
術前	0.877778	79
術前	1.130769	147
術前	1.452174	167
術前	1.866667	280
術前	2.690909	296
術前	2.063636	227
術前	7.653333	574
術前	5.306667	398
術前	3.957143	277
術前	2.166667	260
術前	2.55	306
術前	1.324503	400
術前	5.466667	410
術前	3.809524	400
術前	3.8	380
術前	1.405405	260
術前	1.034483	150
術前	1.538462	200
術前	1.428571	150

術前	2.469136	200
術前	2.403846	250
術前	2.777778	250
術前	2.777778	250
術前	1.25	150
術前	0.572727	63
術前	0.322222	58
術前	1.5	90
術前	1.454545	160
術前	2.8	140
術前	12.66667	190
術前	4.444444	200
術前	8.333333	150
術前	20	140
術前	20	140
術前	11.25	90
術前	1.320755	70
術前	8.333333	100
術前	7.5	90
術前	6.363636	70
術前	3.125	50
術前	0.612245	30
術前	0.625	20
術前	4.444444	400
術前	0.857143	150
術前	2.222222	100
術前	2.857143	100
術前	2.666667	200
術前	4.285714	150
術前	2.666667	200
術前	0.8	100
術前	2.222222	100
術前	0.869565	100
術前	0.869565	200
術前	3.184615	207
術前	2.50838	449
術前	1.375	407
術前	2.928	366
術前	0.489796	240
術前	6	300
術前	3.5	350
術前	2.083333	250
術前	2.777778	250
術前	1.142857	160
術前	0.8	200
術前	3.714286	260
術前	8	480
術前	7.5	225
術前	2.957143	207
術前	1.977778	178
術前	0.936842	178
術前	0.242424	80
術前	0.161905	34
術前	2.8	210
術前	0.807018	230
術前	0.964706	164
術前	1.952	244
術前	2.005405	371
術前	2.571429	270

術前	0.836364	138	
術前	0.823529	140	
術前	3.6875	295	
術前	1.8875	151	
術前	1.35	135	
術前	1.571429	110	
術前	1.75	175	
術前	2.9625	237	
術前	1.115385	145	
術前	0.9	81	
術前	0.857143	270	
術前	1.690909	186	
術前	0.6	276	
術前	0.818182	90	
術前	0.8	80	
術前	0.833333	50	
術前	1.388889	125	
術前	7	140	
術前	3.75	150	
術前	1.538462	100	
術前	2.266667	170	
術前	6.5	130	
術前	3.5	140	
術前	3	120	
術前	1.1	110	
術前	0.882353	150	
3か月	0.769231		50
3か月	0.75		30
3か月	0.833333		50
3か月	0.909091		50
3か月	0.380952		40
3か月	1.052632		100
3か月	1.666667		100
3か月	1.25		100
3か月	1		80
3か月	0.75		30
3か月	0.714286		50
3か月	0.833333		50
3か月	0.8		40
3か月	1		50
3か月	0.705882		120
3か月	1.5		90
3か月	1.25		50
3か月	2.4		120
3か月	3.333333		100
3か月	1.428571		100
3か月	0.75		90
3か月	1.5		90
3か月	1		80
3か月	1.111111		100
3か月	1.428571		100
3か月	1.2		120
3か月	2		200
3か月	3.333333		200
3か月	1.111111		200
3か月	1.176471		200
3か月	1.034483		150
3か月	1.384615		180
3か月	2.222222		200

3か月	2.25	180
3か月	0.944444	170
3か月	0.8	200
3か月	0.863636	190
3か月	1.105263	210
3か月	1.636364	180
3か月	0.333333	100
3か月	1.351351	250
3か月	1.190476	250
3か月	2.142857	300
3か月	2.923077	190
3か月	3	300
3か月	1.142857	200
3か月	0.909091	250
3か月	2.105263	200
3か月	1.73913	200
3か月	1.290323	200
3か月	1.132075	300
3か月	0.363636	20
3か月	0.432432	80
3か月	0.666667	60
3か月	1.111111	100
3か月	1.111111	100
3か月	0.888889	80
3か月	0.526316	100
3か月	1.5	180
3か月	0.47619	200
3か月	1.129032	350
3か月	0.833333	50
3か月	1.125	90
3か月	0.72	90
3か月	0.769231	100
3か月	1.538462	100
3か月	1.692308	110
3か月	0.923077	120
3か月	0.769231	50
3か月	0.833333	100
3か月	1.666667	200
3か月	1.111111	100
3か月	1.846154	120
3か月	4	300
3か月	2.272727	250
3か月	1.090909	120
3か月	0.769231	100
3か月	1.666667	100
3か月	0.833333	100
3か月	1.25	100
3か月	1.428571	100
3か月	0.833333	100
3か月	1.111111	100
3か月	1.111111	100
3か月	0.833333	100
3か月	1.111111	100
3か月	1.111111	100
3か月	1.6	120
3か月	1.266667	190
3か月	1.714286	180
3か月	1.375	110
3か月	0.923077	120

3か月	0.952381	100
3か月	3.125	250
3か月	3.333333	300
3か月	1.785714	250
3か月	2.5	250
3か月	4	200
3か月	2.272727	250
3か月	2.5	200
3か月	2.777778	250
3か月	2.5	200
3か月	3.571429	250
3か月	1.333333	200
3か月	1.666667	300
3か月	1.333333	200
3か月	0.714286	100
3か月	0.5	150
3か月	2.666667	200
3か月	2.857143	200
3か月	4.4	220
3か月	3.636364	200
3か月	2.571429	180
3か月	1.6	160
3か月	1.36	170
3か月	1.578947	150
3か月	0.928571	130
3か月	1.307692	170
3か月	1	70
3か月	0.5	70
3か月	1.428571	100
3か月	1.166667	70
3か月	0.842105	80
3か月	0.823529	70
3か月	0.666667	80
3か月	0.909091	100
3か月	0.529412	90
3か月	0.928571	130
3か月	0.555556	100
3か月	0.652174	150
3か月	0.705882	120
3か月	0.487805	100
3か月	0.914286	160
3か月	0.833333	100
3か月	0.466667	70
3か月	0.888889	80
3か月	0.833333	100
3か月	1	150
3か月	0.25	50
3か月	1.272727	140
3か月	1	160
3か月	1.75	140
3か月	2.333333	140
3か月	2	120
3か月	0.666667	100
3か月	0.909091	100
3か月	3	120
3か月	2.272727	250
3か月	5	100
3か月	1.666667	300
3か月	0.714286	100

3か月	1.111111	100
3か月	0.909091	200
3か月	0.909091	100
3か月	0.526316	50
3か月	1.818182	100
3か月	2	110
3か月	1.538462	100
3か月	2.166667	130
3か月	0.689655	100
3か月	0.555556	50
3か月	0.555556	50
3か月	0.5	50
3か月	0.454545	50
3か月	0.833333	50
3か月	3.238095	340
3か月	5	350
3か月	2.428571	340
3か月	5.555556	500
3か月	2.5	250
3か月	3.625	290
3か月	2.5	400
3か月	5	400
3か月	1	150
3か月	1.25	150
3か月	1.444444	130
3か月	1.222222	110
3か月	1.444444	130
3か月	4.333333	130
3か月	1.25	150
3か月	1.5	180
3か月	1.666667	200
3か月	0.290323	45
3か月	5.5	55
3か月	0.461538	30
3か月	1.666667	100
3か月	2.083333	250
3か月	2.333333	350
3か月	2.777778	250
3か月	3.333333	400
3か月	6	300
3か月	2.5	200
3か月	5.066667	380
3か月	4	220
3か月	2	270
3か月	1	100
3か月	1.894737	180
3か月	1.125	180
3か月	1.058824	180
3か月	4.166667	250
3か月	2	120
3か月	8.333333	250
3か月	3.571429	250
3か月	3.285714	230
3か月	3.333333	200
3か月	1.583333	190
3か月	1.428571	100
3か月	0.555556	50
3か月	2.857143	200
3か月	1.428571	200

3か月	1.923077	250
3か月	1.315789	250
3か月	1.428571	200
3か月	2	200
3か月	1.047619	110
3か月	0.947368	180
3か月	1.461538	190
3か月	1.153846	150
3か月	1.391304	160
3か月	1.111111	250
3か月	0.714286	50
3か月	0.5	40
3か月	0.833333	100
3か月	0.833333	100
3か月	1.428571	100
3か月	0.714286	100
3か月	0.75	90
3か月	1	50
3か月	1.25	50
3か月	6.25	250
3か月	0.588235	100
3か月	1.875	150
3か月	1.521739	350
3か月	1.666667	200
3か月	1	100
3か月	10	100
3か月	0.769231	100
3か月	1.111111	100
3か月	0.833333	100
3か月	0.666667	100
3か月	0.1	30
3か月	1.37931	200
3か月	1.36	170
3か月	2.181818	240
3か月	2.142857	300
3か月	1.857143	260
3か月	1.086957	250
3か月	1.071429	300
3か月	3.125	250
3か月	3.809524	400
3か月	2.857143	300
3か月	0.952381	200
3か月	1.666667	200
3か月	1.25	150
3か月	0.909091	100
3か月	2	80
3か月	4.285714	150
3か月	4.571429	160
3か月	2.142857	150
3か月	1.6	120
3か月	1.636364	90
3か月	1	150
3か月	0.846154	110
3か月	0.75	60
3か月	1	100
3か月	1.181818	130
3か月	1.111111	250
3か月	1.619048	170
3か月	1.238095	130

3か月	2.916667	350	
3か月	2.121212	350	
3か月	1.041667	250	
3か月	1.818182	200	
3か月	2.5	250	
3か月	1.666667	150	
3か月	1.111111	200	
3か月	1	170	
3か月	0.685714	120	
3か月	1.043478	120	
3か月	1.545455	170	
3か月	1.4	140	
3か月	0.566038	150	
3か月	0.387097	60	
3か月	0.555556	50	
3か月	1	120	
3か月	0.833333	100	
3か月	0.944444	170	
3か月	1.083333	130	
3か月	1.333333	160	
3か月	0.365854	150	
3か月	3.142857	110	
3か月	5	150	
3か月	9.333333	140	
3か月	7.5	150	
3か月	1.818182	100	
3か月	2	100	
3か月	0.666667	100	
3か月	0.909091	100	
3か月	0.666667	100	
3か月	0.5	120	
3か月	0.333333	90	
3か月	1.111111	100	
3か月	0.166667	30	
12か月	0.8		120
12か月	1.5		180
12か月	1.666667		250
12か月	2		180
12か月	1.333333		120
12か月	2.222222		200
12か月	3.333333		200
12か月	1.666667		200
12か月	1.666667		200
12か月	1.666667		300
12か月	3.571429		250
12か月	1.388889		250
12か月	1.666667		250
12か月	3.076923		200
12か月	0.4		20
12か月	1.842105		350
12か月	1.130435		260
12か月	1.2		180
12か月	1.388889		250
12か月	1.083333		260
12か月	1		120
12か月	2.285714		320
12か月	3.571429		250
12か月	4		240
12か月	2		300

12か月	1.083333	130
12か月	1.689189	250
12か月	1.470588	250
12か月	0.677966	200
12か月	2.173913	250
12か月	1	250
12か月	1.935484	300
12か月	1.666667	150
12か月	1.041667	100
12か月	5	180
12か月	6.341463	260
12か月	4.545455	200
12か月	3.08642	250
12か月	1.709402	200
12か月	1.388889	200
12か月	1.415094	150
12か月	1.984127	250
12か月	3.45679	280
12か月	0.871795	170
12か月	1.666667	150
12か月	1.714286	120
12か月	3.4	170
12か月	6	240
12か月	2.352941	200
12か月	4.363636	240
12か月	6.25	250
12か月	0.888889	80
12か月	2	200
12か月	1.481481	200
12か月	1.904762	200
12か月	0.952381	200
12か月	1.333333	200
12か月	0.909091	200
12か月	0.736842	70
12か月	0.588235	50
12か月	1	110
12か月	1.555556	140
12か月	2.5	100
12か月	0.294118	50
12か月	0.526316	100
12か月	0.833333	100
12か月	2.222222	200
12か月	1.666667	150
12か月	2.333333	350
12か月	6.666667	400
12か月	2.916667	350
12か月	2.222222	200
12か月	1.388889	250
12か月	1.111111	100
12か月	1.428571	200
12か月	1.5	150
12か月	1.666667	200
12か月	1.666667	200
12か月	1.666667	150
12か月	1.333333	200
12か月	1.25	150
12か月	1.666667	200
12か月	1.5	180
12か月	0.714286	150

12か月	1.6	160
12か月	1.8	180
12か月	1.391304	160
12か月	1.904762	200
12か月	1.315789	250
12か月	1.153846	150
12か月	1.5	150
12か月	1.538462	200
12か月	2.142857	150
12か月	1.666667	150
12か月	1.538462	200
12か月	0.882353	150
12か月	7.5	150
12か月	0.588235	100
12か月	0.833333	100
12か月	0.833333	150
12か月	0.666667	100
12か月	0.833333	100
12か月	0.416667	50
12か月	2	220
12か月	2.25	270
12か月	1.84	230
12か月	1.212121	200
12か月	2.545455	280
12か月	1.785714	250
12か月	1.636364	180
12か月	0.434783	100
12か月	0.30303	100
12か月	0.4	50
12か月	0.3125	100
12か月	0.923077	120
12か月	0.740741	100
12か月	0.731707	150
12か月	1.090909	180
12か月	0.758621	220
12か月	0.555556	50
12か月	0.555556	50
12か月	0.666667	100
12か月	0.888889	80
12か月	0.512821	100
12か月	0.526316	150
12か月	1	120
12か月	1.166667	140
12か月	1.5	120
12か月	1.75	140
12か月	0.681818	150
12か月	1.166667	140
12か月	1.818182	200
12か月	2.5	200
12か月	1.612903	250
12か月	1.153846	150
12か月	0.555556	50
12か月	1.136364	250
12か月	2.352941	200
12か月	2.222222	200
12か月	3.333333	200
12か月	1.25	150
12か月	0.47619	100
12か月	0.416667	50

12か月	0.588235	100
12か月	1.666667	250
12か月	4.285714	300
12か月	2.5	250
12か月	2.307692	300
12か月	1.333333	200
12か月	1.315789	250
12か月	3.181818	350
12か月	1.666667	200
12か月	0.833333	200
12か月	1.111111	200
12か月	1.111111	200
12か月	1.111111	200
12か月	1.666667	200
12か月	0.833333	100
12か月	0.315789	30
12か月	0.416667	50
12か月	0.62069	180
12か月	0.588235	50
12か月	5	300
12か月	4	320
12か月	1.307692	170
12か月	1.133333	170
12か月	2.2	330
12か月	2.083333	250
12か月	1.666667	200
12か月	2.5	300
12か月	2.285714	80
12か月	1.538462	200
12か月	0.8	180
12か月	1.190476	250
12か月	1.333333	200
12か月	0.606061	100
12か月	0.888889	80
12か月	1.818182	200
12か月	2.142857	150
12か月	2.857143	300
12か月	1.666667	250
12か月	1.52381	160
12か月	1.7	170
12か月	2	140
12か月	1.818182	200
12か月	2.142857	150
12か月	1.25	250
12か月	0.645161	200
12か月	0.441176	150
12か月	5	300
12か月	0.277778	50
12か月	0.833333	50
12か月	0.714286	50
12か月	1.111111	200
12か月	1.111111	100
12か月	1.111111	150
12か月	5.714286	200
12か月	1.304348	150
12か月	5.142857	360
12か月	1.958333	470
12か月	1.125	360
12か月	1	200

12か月	2.5	300
12か月	2.142857	300
12か月	1.25	200
12か月	1.538462	400
12か月	1.117647	190
12か月	2.307692	150
12か月	2.888889	130
12か月	2.75	110
12か月	2.5	100
12か月	7.2	180
12か月	1.294118	110
12か月	1.333333	100
12か月	0.909091	100
12か月	0.8	120
12か月	1.176471	100
12か月	2.571429	180
12か月	0.555556	100
12か月	6	180
12か月	0.666667	120
12か月	1.666667	250
12か月	0.952381	200
12か月	1.666667	200
12か月	1.666667	250
12か月	1.190476	250
12か月	0.588235	150
12か月	0.731707	150
12か月	1.351351	250
12か月	0.666667	150
12か月	0.555556	100
12か月	0.607143	170
12か月	0.611111	110
12か月	3.454545	380
12か月	0.47619	50
12か月	1	120
12か月	1.666667	100
12か月	1.25	150
12か月	1.666667	100
12か月	1.25	150
12か月	1	120
12か月	1.5	180
12か月	0.792453	210
12か月	1.043478	120
12か月	2.4	180
12か月	1.066667	80
12か月	2.727273	300
12か月	1.9	190
12か月	0.75	120

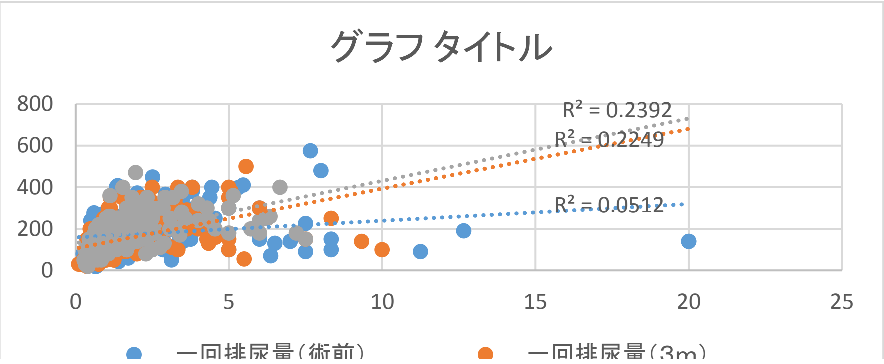

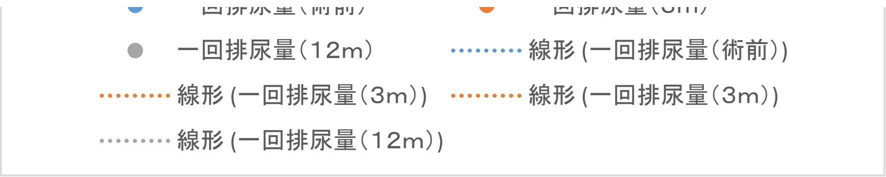
一回排尿量（12m）
線形 (一回排尿量（術前）)
線形 (一回排尿量（3m）)
線形 (一回排尿量（3m）)
線形 (一回排尿量（12m）)
