# Supplementary material for: The Effect of the Vesical Adaptation Response to Diuresis on Lower Urinary Tract Symptoms after Robot-Assisted Laparoscopic Radical Prostatectomy: A Pilot Proof of Concept Study
Source: PLoS One. 2016 Jul 22;11(7):e0159514. doi: 10.1371/journal.pone.0159514 (PMC4957788; doi:10.1371/journal.pone.0159514)

調査時期	利尿率	一回排尿	一回排尿	一回排尿量（12か月）
術前	2.444444	220		
術前	1.73913	200		
術前	0.869565	100		
術前	0.947368	180		
術前	1.136364	250		
術前	1.081081	400		
術前	2.727273	450		
術前	5.333333	400		
術前	2.25	450		
術前	0.871429	244		
術前	2.271429	477		
術前	3.504348	403		
術前	2.255172	327		
術前	0.917241	266		
術前	0.689655	300		
術前	1.25	300		
術前	2.222222	400		
術前	1.142857	200		
術前	0.782609	180		
術前	3.463158	329		
術前	2.5375	406		
術前	1.745098	445		
術前	3.966667	357		
術前	1.175	329		
術前	0.816667	98		
術前	1.138095	239		
術前	3.066667	460		
術前	1.08	324		
術前	1	220		
術前	1.25	50		
術前	2.833333	170		
術前	1.666667	200		
術前	2.77037	374		
術前	0.869565	100		
術前	1	115		
術前	2.142857	150		
術前	3.333333	250		
術前	2.083333	250		
術前	0.774194	120		
術前	1	100		
術前	1.384	346		
術前	1.590244	326		
術前	1.252381	263		
術前	0.588235	100		
術前	3.231579	307		
術前	1.914286	201		
術前	3.5	210		
術前	0.909091	200		
術前	0.75	120		
術前	2	200		
術前	2	250		
術前	1.666667	250		
術前	0.307692	60		
術前	0.536842	153		
術前	1.477419	229		
術前	2.233333	402		
術前	1.308333	157		
術前	1.071429	150		

術前	2.5	150
術前	2.347826	270
術前	2	230
術前	2.777778	250
術前	1.935484	300
術前	1.666667	300
術前	0.464	116
術前	2	180
術前	6.2	310
術前	3.5	350
術前	0.9375	300
術前	0.952381	100
術前	2.118644	250
術前	4.123711	400
術前	4.444444	400
術前	1.212121	200
術前	2.222222	100
術前	1.086957	250
術前	3.125	250
術前	3	300
術前	0.735294	250
術前	1.052632	200
術前	1.666667	300
術前	3.333333	500
術前	1.4	420
術前	1.973333	296
術前	2.142857	300
術前	1.333333	320
術前	0.2	40
術前	1.428571	400
術前	3.125	250
術前	1.230769	80
術前	1.111111	150
術前	1.071429	150
術前	1	80
術前	1.6	200
術前	2.266667	170
術前	0.545455	150
術前	2.702703	500
術前	1.090909	300
術前	2.068966	300
術前	1.6	200
術前	5.063291	400
術前	1.724138	200
術前	0.610526	174
術前	2.345455	129
術前	2.3625	189
術前	2.649485	257
術前	3.380435	311
術前	1.68125	269
術前	1.45	290
術前	0.909091	150
術前	2.4	300
術前	1.666667	200
術前	2.857143	200
術前	1.666667	200
術前	1.666667	250
術前	1.785714	250
術前	1.314286	230

術前	2.666667	240
術前	4	280
術前	2.076923	270
術前	1.411765	240
術前	0.645161	200
術前	2.142857	300
術前	2.727273	150
術前	0.952381	300
術前	0.25641	50
術前	1.166667	350
術前	0.833333	200
術前	0.591667	142
術前	3.6375	291
術前	6.633333	398
術前	6.290909	346
術前	4.228571	296
術前	2.617391	301
術前	1.138095	239
術前	1.470588	200
術前	1.666667	280
術前	2.409639	200
術前	2	200
術前	1.515152	200
術前	2.777778	200
術前	1.747573	180
術前	2.631579	100
術前	1.666667	200
術前	3.125	250
術前	1.25	150
術前	1.987952	330
術前	4.761905	400
術前	4.583333	275
術前	2.083333	125
術前	1.666667	250
術前	0.789474	150
術前	1.25	150
術前	1.666667	150
術前	2.2	330
術前	2.233333	201
術前	2.834783	326
術前	2.264516	351
術前	1.958621	284
術前	0.833333	200
術前	2	150
術前	1.111111	200
術前	1.025641	200
術前	1.111111	200
術前	1.666667	200
術前	0.726496	170
術前	0.666667	150
術前	0.952381	100
術前	1.458333	350
術前	1.71	171
術前	2.417647	411
術前	2.431579	231
術前	2	310
術前	0.857955	302
術前	1.704918	312
術前	3.452632	328

術前	0.853333	192	
術前	1.333333	200	
術前	3.125	250	
術前	2.111111	190	
術前	2.225	267	
術前	3.5	245	
術前	1.111111	150	
術前	4.888889	220	
術前	0.731707	150	
術前	1.315789	250	
術前	3.692308	240	
術前	3.333333	200	
術前	1.666667	300	
術前	1.666667	200	
術前	0.857143	150	
術前	1.376384	373	
術前	3.076923	320	
術前	1.612245	316	
術前	2.662162	394	
術前	2.909091	320	
術前	0.689655	200	
術前	1.028571	180	
術前	2.25	180	
術前	1.703704	230	
術前	1.176471	200	
術前	0.921053	175	
術前	1.666667	350	
術前	0.952381	200	
術前	2.666667	400	
3か月	1.571429		110
3か月	1.111111		100
3か月	1.5		120
3か月	1.714286		120
3か月	2		100
3か月	1.875		150
3か月	1.333333		120
3か月	1.714286		120
3か月	1.222222		110
3か月	1.166667		140
3か月	1.571429		110
3か月	2.4		120
3か月	1		100
3か月	1.454545		80
3か月	1.294118		110
3か月	2.5		200
3か月	1.666667		200
3か月	1.875		300
3か月	0.25		20
3か月	0.833333		50
3か月	1.555556		70
3か月	0.666667		50
3か月	1		40
3か月	1.333333		200
3か月	0.396825		250
3か月	1.176471		200
3か月	1.304348		150
3か月	1.666667		350
3か月	7.5		300
3か月	5.555556		500

3か月	2.272727	250
3か月	2	300
3か月	1.785714	250
3か月	1.875	150
3か月	1	180
3か月	0.588235	150
3か月	2.769231	180
3か月	0.888889	120
3か月	1.025641	200
3か月	1	50
3か月	0.714286	100
3か月	1.363636	150
3か月	1.764706	150
3か月	1.111111	50
3か月	2.142857	150
3か月	0.833333	50
3か月	2	100
3か月	1.454545	80
3か月	1.111111	100
3か月	1.111111	200
3か月	1.851852	250
3か月	1.5	210
3か月	1.384615	180
3か月	0.88	220
3か月	1.307692	170
3か月	0.863636	190
3か月	1.333333	120
3か月	0.571429	100
3か月	0.491803	150
3か月	0.648649	120
3か月	1.020408	250
3か月	1	150
3か月	1.428571	200
3か月	1.176471	**300**
3か月	1.944444	**350**
3か月	1.428571	**250**
3か月	1.388889	**250**
3か月	1.25	**100**
3か月	1.666667	50
3か月	0.666667	20
3か月	0.777778	70
3か月	0.666667	100
3か月	0.666667	20
3か月	0.333333	20
3か月	0.333333	100
3か月	0.4	50
3か月	0.566667	51
3か月	0.615385	40
3か月	0.622222	28
3か月	0.382857	67
3か月	0.616667	37
3か月	0.8	40
3か月	0.57	57
3か月	0.333333	25
3か月	0.282353	24
3か月	1.565217	180
3か月	1.851852	250
3か月	1.555556	280
3か月	0.681818	150

3か月	3.333333	200
3か月	1.75	70
3か月	1.5	120
3か月	0.916667	110
3か月	1	150
3か月	0.684211	130
3か月	1.235294	210
3か月	1.571429	220
3か月	1.333333	120
3か月	1.181818	130
3か月	0.846154	110
3か月	1.066667	80
3か月	1.185185	160
3か月	0.833333	100
3か月	0.833333	50
3か月	0.555556	50
3か月	0.833333	100
3か月	0.47619	100
3か月	2.592593	350
3か月	2.8	350
3か月	0.833333	350
3か月	3.571429	250
3か月	1.407407	190
3か月	1.428571	150
3か月	0.727273	120
3か月	2.571429	180
3か月	2.4	120
3か月	1.333333	120
3か月	0.8	80
3か月	2.2	220
3か月	10	300
3か月	7.5	300
3か月	1.5	150
3か月	0.666667	100
3か月	2	120
3か月	0.714286	100
3か月	2.857143	300
3か月	1.538462	100
3か月	0.909091	150
3か月	2.352941	200
3か月	10	300
3か月	12	300
3か月	0.166667	20
3か月	0.5	85
3か月	0.854167	410
3か月	1.043478	120
3か月	0.952381	200
3か月	1.481481	200
3か月	1.052632	100
3か月	1.304348	150
3か月	0.952381	100
3か月	0.833333	50
3か月	0.666667	100
3か月	1	150
3か月	1	150
3か月	1.666667	100
3か月	2.5	200
3か月	1.538462	200
3か月	1.25	100

3か月	3.529412	300
3か月	3.333333	100
3か月	1.587302	100
3か月	4.807692	250
3か月	0.793651	250
3か月	0.952381	200
3か月	0.956522	220
3か月	0.590909	130
3か月	1.466667	220
3か月	0.652174	150
3か月	1.133333	170
3か月	2.425532	570
3か月	1.033333	310
3か月	3.333333	400
3か月	2.846154	370
3か月	2.235294	380
3か月	1.6	400
3か月	2.923077	380
3か月	1.315789	250
3か月	1.545455	170
3か月	1.095238	230
3か月	1	150
3か月	0.322581	100
3か月	1	60
3か月	1.176471	300
3か月	2.380952	250
3か月	2.222222	200
3か月	2.083333	250
3か月	2.222222	200
3か月	0.833333	100
3か月	0.8	120
3か月	0.432432	80
3か月	1	120
3か月	0.787879	130
3か月	2	150
3か月	1.8	180
3か月	1.875	150
3か月	0.833333	100
3か月	0.842105	80
3か月	1.181818	130
3か月	1.636364	180
3か月	0.733333	110
3か月	6.666667	200
3か月	4.210526	400
3か月	2	50
3か月	4.444444	200
3か月	0.882353	150
3か月	0.833333	250
3か月	1.142857	200
3か月	1.666667	250
3か月	0.416667	50
3か月	0.666667	100
3か月	1.538462	100
3か月	2.105263	400
3か月	0.434783	50
3か月	0.769231	50
3か月	0.909091	150
3か月	1	150
3か月	0.833333	150

3か月	0.277778	50
3か月	0.666667	100
3か月	1.666667	250
3か月	0.909091	50
3か月	0.941176	80
3か月	0.727273	80
3か月	0.222222	20
3か月	0.444444	20
3か月	0.869565	100
3か月	0.32	40
3か月	1.25	100
3か月	1.454545	160
3か月	0.684211	65
3か月	0.456522	105
3か月	1.117647	95
3か月	1.230769	80
3か月	0.301887	80
3か月	0.171429	30
3か月	2.222222	200
3か月	2	200
3か月	2	200
3か月	1.481481	200
3か月	1.142857	200
3か月	1.888889	170
3か月	2	180
3か月	2.375	190
3か月	0.833333	100
3か月	0.848485	140
3か月	0.431373	110
3か月	0.444444	100
3か月	2	100
3か月	1.25	150
3か月	2	100
3か月	1.571429	110
3か月	1.071429	150
3か月	2.142857	150
3か月	1.142857	120
3か月	1.25	150
3か月	0.742857	130
3か月	1.818182	**300**
3か月	1.282051	**250**
3か月	1.944444	**350**
3か月	1.666667	**400**
3か月	1.666667	**400**
3か月	0.9375	150
3か月	0.588235	100
3か月	0.666667	100
3か月	0.662983	120
3か月	1.492537	200
3か月	1.363636	150
3か月	1.428571	150
3か月	1.388889	250
3か月	2.222222	100
3か月	6.153846	400
3か月	6.25	250
3か月	1.904762	200
3か月	1.666667	150
3か月	1.041667	250
3か月	2.142857	300

3か月	3.076923	400	
3か月	1.851852	250	
3か月	1.428571	150	
3か月	1.666667	150	
3か月	1.428571	300	
3か月	1.714286	**180**	
3か月	2.857143	**200**	
3か月	6.666667	**200**	
3か月	3.538462	**230**	
3か月	3.692308	**240**	
3か月	2.75	**220**	
3か月	2.25	**180**	
3か月	4	**240**	
3か月	1.666667	**150**	
3か月	1.333333	**160**	
3か月	0.208333	100	
3か月	1	120	
3か月	1.3	130	
3か月	0.357143	50	
3か月	1.25	200	
3か月	0.555556	50	
3か月	1.333333	200	
3か月	0.952381	100	
3か月	0.888889	200	
3か月	0.333333	50	
3か月	0.666667	100	
3か月	1.666667	250	
3か月	1.916667	230	
3か月	1.25	150	
3か月	1.333333	200	
3か月	2.75	165	
3か月	1.666667	200	
3か月	1.818182	200	
3か月	0.833333	100	
3か月	0.761905	80	
3か月	0.375	90	
3か月	0.625	100	
3か月	0.636364	70	
3か月	0.555556	50	
3か月	1.052632	200	
3か月	1.285714	180	
3か月	1.333333	240	
3か月	0.833333	150	
3か月	0.666667	100	
3か月	2.857143	200	
3か月	1.190476	250	
3か月	0.666667	200	
3か月	0.666667	200	
3か月	2.222222	200	
3か月	0.8	120	
3か月	0.833333	100	
3か月	1.111111	200	
12か月	1.4		140
12か月	0.882353		150
12か月	1.454545		160
12か月	1.727273		190
12か月	1.636364		180
12か月	1.3		130
12か月	4		200

１２か月	1.315789	250
１２か月	1.384615	180
１２か月	1.090909	120
１２か月	1.076923	140
１２か月	2.4	180
１２か月	1.272727	140
１２か月	1.6	120
１２か月	1.185185	160
１２か月	1.411765	120
１２か月	1.666667	100
１２か月	0.222222	20
１２か月	1.222222	110
１２か月	0.833333	100
１２か月	0.555556	50
１２か月	1	70
１２か月	0.578947	110
１２か月	0.416667	50
１２か月	0.666667	**100**
１２か月	1.287879	**425**
１２か月	1.025641	**400**
１２か月	0.833333	**50**
１２か月	1.509434	400
１２か月	5.333333	400
１２か月	6.428571	450
１２か月	8.333333	500
１２か月	3.032258	470
１２か月	2.5	200
１２か月	2.173913	250
１２か月	0.769231	100
１２か月	1.875	300
１２か月	1.515152	250
１２か月	1.785714	250
１２か月	1.538462	300
１２か月	0.540541	200
１２か月	1.25	200
１２か月	0.943396	250
１２か月	0.878049	180
１２か月	2.842105	270
１２か月	2.066667	310
１２か月	1.5625	250
１２か月	1.52381	320
１２か月	1.45	290
１２か月	1.857143	260
１２か月	1.310345	190
１２か月	1.125	90
１２か月	0.225352	80
１２か月	0.583333	70
１２か月	1.388889	250
１２か月	0.727273	120
１２か月	1.764706	300
１２か月	1.818182	**300**
１２か月	1.333333	**300**
１２か月	1.052632	**200**
１２か月	4.666667	**700**
１２か月	9.166667	**550**
１２か月	0.142857	30
１２か月	0.214286	30
１２か月	0.8	100
１２か月	1.818182	100

１2か月	0.454545	50
１2か月	2	130
１2か月	4	100
１2か月	0.714286	150
１2か月	0.571429	100
１2か月	0.769231	200
１2か月	0.4	100
１2か月	0.333333	20
１2か月	0.727273	40
１2か月	3.555556	160
１2か月	1.875	150
１2か月	1.714286	120
１2か月	1.214286	170
１2か月	1.090909	120
１2か月	2.142857	150
１2か月	0.8	120
１2か月	2.285714	160
１2か月	1.363636	150
１2か月	0.8	60
１2か月	1	180
１2か月	0.8	120
１2か月	1.25	200
１2か月	0.714286	100
１2か月	0.5	150
１2か月	1.071429	300
１2か月	1.481481	400
１2か月	3.333333	300
１2か月	1.368421	130
１2か月	1.241379	180
１2か月	0.529412	90
１2か月	0.864865	160
１2か月	0.947368	90
１2か月	1.583333	190
１2か月	1.666667	100
１2か月	2.916667	350
１2か月	6.666667	300
１2か月	1.428571	150
１2か月	1.363636	150
１2か月	1.125	180
１2か月	1.818182	300
１2か月	0.909091	150
１2か月	1.25	150
１2か月	1.25	150
１2か月	0.78125	250
１2か月	3.461538	450
１2か月	3.12	390
１2か月	1.294118	330
１2か月	0.888889	200
１2か月	0.971429	170
１2か月	0.784314	200
１2か月	0.545455	120
１2か月	1.571429	220
１2か月	0.909091	150
１2か月	1.333333	200
１2か月	1.666667	300
１2か月	1.333333	200
１2か月	3.333333	200
１2か月	2.5	300
１2か月	2.5	150

12か月	0.540541	300
12か月	0.925926	250
12か月	1.333333	200
12か月	1.2	180
12か月	1.133333	170
12か月	1	150
12か月	1.066667	160
12か月	1.25	150
12か月	3.636364	200
12か月	4.666667	350
12か月	1.333333	260
12か月	1.103448	160
12か月	0.790698	170
12か月	0.923077	120
12か月	0.588235	200
12か月	0.757576	250
12か月	1.166667	280
12か月	1	200
12か月	0.37037	100
12か月	0.952381	200
12か月	0.952381	200
12か月	1.333333	200
12か月	1.2	180
12か月	0.666667	120
12か月	0.833333	150
12か月	1.166667	210
12か月	2.5	150
12か月	1.666667	100
12か月	0.6	90
12か月	0.533333	80
12か月	0.592593	80
12か月	0.615385	120
12か月	1.235294	210
12か月	0.75	30
12か月	1.333333	200
12か月	1	150
12か月	0.740741	200
12か月	1.428571	150
12か月	2	150
12か月	0.666667	100
12か月	1	50
12か月	0.808511	190
12か月	0.530612	130
12か月	1.818182	200
12か月	5.75	230
12か月	10	300
12か月	2.933333	220
12か月	0.666667	30
12か月	1.5625	250
12か月	1.428571	200
12か月	2.777778	250
12か月	1.111111	200
12か月	1.153846	300
12か月	1.538462	200
12か月	0.289855	200
12か月	0.833333	200
12か月	0.833333	200
12か月	0.666667	200
12か月	0.5	150

１２か月	1.612903	250
１２か月	0.909091	100
１２か月	0.857143	60
１２か月	1.111111	100
１２か月	0.666667	50
１２か月	0.666667	100
１２か月	0.909091	100
１２か月	1.153846	150
１２か月	0.684211	130
１２か月	0.652174	150
１２か月	0.631579	120
１２か月	1.230769	80
１２か月	0.311111	70
１２か月	1.071429	150
１２か月	1.73913	200
１２か月	1.111111	150
１２か月	2.272727	250
１２か月	2.5	200
１２か月	0.727273	160
１２か月	0.625	100
１２か月	3.571429	**500**
１２か月	1.428571	**500**
１２か月	1.75	**350**
１２か月	0.5	80
１２か月	0.555556	100
１２か月	0.909091	100
１２か月	0.571429	80
１２か月	0.393443	120
１２か月	1.111111	**300**
１２か月	2	**300**
１２か月	1.73913	**200**
１２か月	1.481481	**200**
１２か月	1.166667	**350**
１２か月	1.058824	180
１２か月	1.909091	210
１２か月	3.2	400
１２か月	1.818182	300
１２か月	1.142857	80
１２か月	2.25	180
１２か月	1	120
１２か月	1.025641	200
１２か月	1.818182	300
１２か月	0.510638	120
１２か月	1.111111	100
１２か月	1.666667	100
１２か月	2	180
１２か月	3.333333	300
１２か月	6	300
１２か月	2.142857	150
１２か月	1.111111	100
１２か月	1.25	50
１２か月	1.5	150
１２か月	1.428571	200
１２か月	2.222222	200
１２か月	3.857143	270
１２か月	2.439024	200
１２か月	5.510204	270
１２か月	2.738095	230
１２か月	2.090909	230

12か月	2.3	230
12か月	2.941176	250
12か月	1.1	220
12か月	0.740741	100
12か月	1.071429	150
12か月	1.714286	120
12か月	1.777778	240
12か月	1.25	200
12か月	1.304348	150
12か月	1.666667	200
12か月	0.416667	100
12か月	1	150
12か月	1.111111	100
12か月	0.846154	110
12か月	1.666667	250
12か月	1.153846	150
12か月	1.428571	350
12か月	0.769231	150
12か月	1.333333	280
12か月	0.555556	50
12か月	2.8	280
12か月	2.5	250
12か月	2.5	250
12か月	1.52381	320
12か月	1.047619	220
12か月	4.181818	230
12か月	2.933333	220
12か月	0.875	210
12か月	0.909091	200
12か月	0.55814	120
12か月	0.555556	100
12か月	1	150
12か月	2.4375	390
12か月	0.576923	150
12か月	1.6	240
12か月	0.851852	230
12か月	1.222222	220
12か月	0.623557	270
12か月	0.833333	150
12か月	1.041667	250
12か月	0.833333	100

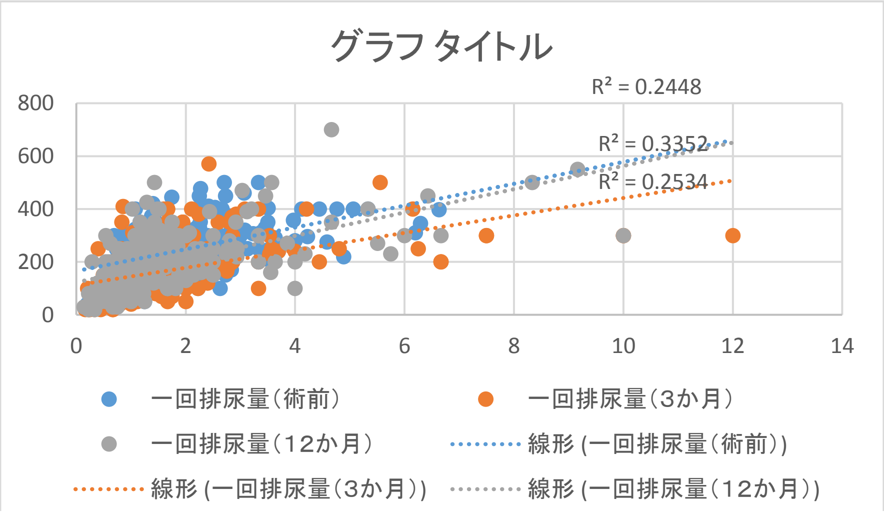

Supplement: S2 File — The patients’ clinical parameters, which was recorded in the patients without preoperative LUTS, were described. (PDF) [file pone.0159514.s002.pdf]
